# Supplementary material for: The effects of polygenic risk for psychiatric disorders and smoking behaviour on psychotic experiences in UK Biobank
Source: Transl Psychiatry. 2020 Sep 28;10:330. doi: 10.1038/s41398-020-01009-8 (PMC7523004; doi:10.1038/s41398-020-01009-8)
Supplement: Supplementary file 1 — Supplemental material [file 41398_2020_1009_MOESM1_ESM.docx]

**Supplementary Materials for:**

*The effects of polygenic risk for psychiatric disorders and smoking behaviour on psychotic experiences in UK Biobank*

**Supplementary Tables**

| Page 2 - | **Supplementary Table 1.** Phenotype definition for lifetime delusions. |
| --- | --- |
| Page 2 - | **Supplementary Table 2.** Phenotype definition for lifetime hallucinations. |
| Page 3 - | **Supplementary Table 3.** Phenotype definition for any lifetime psychotic experience. |
| Page 4 - | **Supplementary Table 4.** Phenotype definition for diagnosis of depression, ADHD and psychotic disorders. |
| Page 6 - | **Supplementary Table 5.** List of antidepressants and antipsychotics used for defining symptoms of depression and probable diagnosis of psychotic disorders. |
| Page 7 - | **Supplementary Table 6.** Number of individuals excluded as part of the quality control of genetic data. |
| Page 8 - | **Supplementary Table 7.** Co-occurrence of smoking behaviours and psychotic experiences after quality control. |
| Page 9 - | **Supplementary Table 8**. Interaction effects between PRSs and smoking status (never vs former vs current) on psychotic experiences (delusions, hallucinations or any of these two). |
| Page 11 - | **Supplementary Table 9.** Number of individuals with psychotic disorders that were excluded. |
| Page 11 - | **Supplementary Table 10**. Interaction effects between PRSs and smoking status (never vs former vs current) on delusions after excluding individuals with psychotic disorders. |
| Page 12 - | **Supplementary Table 11**. Interaction effects between PRSs and maternal smoking on psychotic experiences (delusions, hallucinations or any of these two). |
| Page 13 - | **Supplementary Table 12**. Interaction effects between PRSs and the number of packs smoked per year on psychotic experiences (delusions, hallucinations or any of these two). |

**Supplementary Figures**

| Page 14 - | **Supplementary Figure 1:** Prediction of smoking behaviours in UKB using PRSs for schizophrenia (Panel A), depression (Panel B), ADHD (Panel C) and bipolar disorder (Panel D). |
| --- | --- |
| Page 15 - | **Supplementary Figure 2**. Sensitivity analyses, excluding individuals with diagnosis of psychotic disorders. |

**Supplementary information**

| Page 16 - | **Polygenic score approach** |
| --- | --- |
| Page 17 - | **PRS power calculation** |
| Page 18 - | **Supplementary references** |

**Supplementary Table 1.** Phenotype definition for lifetime delusions.

| **Description** | **Fields and codes** | **N** | **Notes and references** |
| --- | --- | --- | --- |
| **Case: Delusions ever**.  Endorsed possible delusion | Believed unreal conspiracy f.20468 = Yes  OR  Believed unreal communication or signs f.20474 = yes | 2 067 | Code adapted from Davis et al, 2020 ^1^ |
| **Control: Unusual experience ever**. | Believed unreal conspiracy f.20468 = no  AND  Believed unreal communication or signs f.20474 = no | 155 182 |  |
| Number of controls: **155 182 individuals**  Number of cases: **2 067 individuals** | | | |

**Supplementary Table 2.** Phenotype definition for lifetime hallucinations.

| **Description** | **Fields and codes** | **N** | **Notes and references** |
| --- | --- | --- | --- |
| **Case: Hallucinations ever**.  Endorsed possible hallucination | Hear unreal voice f.20463 = yes  OR  Saw unreal vision f.20471 = yes | 6 689 | Code adapted from Davis et al, 2020 ^1^ |
| **Control: Unusual experience ever**. | Hear unreal voice f.20463 = no  AND  Saw unreal vision f.20471 = no | 150 336 |  |
| Number of controls: **150 336 individuals**  Number of cases: **6 689 individuals** | | | |

**Supplementary Table 3.** Phenotype definition for any lifetime psychotic experience.

| **Description** | **Fields and codes** | **N** | **Notes and references** |
| --- | --- | --- | --- |
| **Case: Unusual experience ever**.  Endorsed possible hallucination or delusion | Hear unreal voice f.20463 = yes  OR  Saw unreal vision f.20471 = yes  OR  Believed unreal conspiracy f.20468 = yes  OR  Believed unreal communication or signs f.20474 = yes | 7 803 | Code adapted from Davis et al, 2020 ^1^ |
| **Control: Unusual experience ever**. | Hear unreal voice f.20463 = no  AND  Saw unreal vision f.20471 = no  AND  Believed unreal conspiracy f.20468 = no  AND  Believed unreal communication or signs f.20474 = no  AND  Self-reported physician diagnosis of schizophrenia or any other type of psychosis or psychotic illness during Mental Health Questionnaire interview f.20544 = no | 149 289 |  |
| Number of controls: **149 289 individuals**  Number of cases: **7 803 individuals** | | | |

**Supplementary Table 4.** Phenotype definition for diagnosis of depression, ADHD and psychotic disorders.

| **Origin** | **Description** | **Fields and codes** | **N** | **Notes and references** |
| --- | --- | --- | --- | --- |
| Baseline interview.  Full cohort | Depression  Post-natal depression | For item f.20002, reporting diagnosis of depression (coded=1286), OR post-natal depression (coded=1531) | 28 472 |  |
| Treatment medication code.  Full cohort | Depression | Presence of antidepressants in treatment medication code (f.20003). | 36 786 | Antidepressants in Supplementary Table 5 |
| In-hospital with primary or secondary diagnosis of Mental Health disorders.  Full cohort | Depression | Main and secondary diagnoses from hesin.tsv and hesin_diag10.tsv files.  Included all the items from categories corresponding to depressive episode or recurrent depressive disorder: 'F320','F321','F322','F323','F328','F329','F330','F331','F332','F333','F334','F338','F339' | 4 086 |  |
| Mental Health Questionnaire Screening | Depression (ever) | Persistent sadness (f.20446) = Yes OR Loss of interest (f.20441) = Yes  AND  How much of day (f.20436) = Most of day or All day long  AND  Did you feel this way (f.20439) = Almost every day or every day  AND  Impairment (f.20440) = Somewhat or A lot  AND  Total number of symptoms endorsed (core and others) >= 5  Persistent sadness (core) f.20446; Loss of interest (core) f.20441; Tired or low energy f.20449; Gain or loss of weight f.20536 = Gain, Loss or Gain and loss; Sleep change f.20532; Trouble concentrating f.20435; Feeling worthless f.20450; Thinking about death f.20437 | 37 430 | CIDI-SF (Composite International Diagnostic Interview – Short Form), depression module, lifetime version. Scored based on DSM definition of major depressive disorder ^2^ |
| Mental Health Questionnaire – Self-reported clinician diagnoses | Depression | For item f. 20544 the array of 16 columns screened for: ‘Depression’ | 33 422 |  |
| **Combine Above** | **Lifetime diagnosis of depression** | | **88 976** |  |
| Mental Health Questionnaire – Self-reported clinician diagnoses | ADHD | For item f. 20544 the array of 16 columns screened for diagnosis of Attention deficit or attention deficit and hyperactivity disorder (ADD/ADHD) | 133 |  |
| **Combine Above** | **Lifetime diagnosis of ADHD** | | **133** |  |

| **Origin** | **Description** | **Fields and codes** | **N** |  |
| --- | --- | --- | --- | --- |
| Mental Health Questionnaire | Self-reported physician diagnosis of schizophrenia, mania, hypomania, bipolar or manic depression or any other type of psychotic disorder | Schizophrenia (coded=2 in f.20544) OR  any other type of psychosis or psychotic illness (coded=3 in f.20544) | Schizophrenia N = 157  Any other type of psychosis or psychotic illness N = 604  Mania, hypomania, bipolar or manic-depression N = 837 |  |
| Hospital admissions | Schizophrenia, schizotypal and delusional disorders  OR  Mood [affective] disorders | Main and secondary diagnoses from hesin.tsv and hesin_diag10.tsv files.  **ICD-10 codes:**  'F200','F201','F202','F203','F204','F205','F206','F208','F209', 'F21','F220','F228','F229','F230','F231','F232','F233','F238','F239', 'F24', 'F250','F251','F252','F258','F259','F28','F29','F300','F301','F302','F308','F309','F310','F311','F312','F313','F314','F315','F316','F317','F318','F319' | N = 1 894 | ICD-10 codes obtained from UK Biobank: <http://biobank.ctsu.ox.ac.uk/crystal/field.cgi?id=41202> |
| Baseline assessment centre | Self-reported diagnosis of schizophrenia or mania/bipolar disorder/ manic depression in oral interview | Reporting diagnosis of schizophrenia (coded=1289) OR mania/bipolar disorder/ manic depression (coded=1291) for item f.20002 | N = 1 995 |  |
| Death records | Schizophrenia, schizotypal and delusional disorders  OR  Mood [affective] disorders | Main and secondary causes of death from death record files  **ICD-10 codes:**  'F200','F201','F202','F203','F204','F205','F206','F208','F209', 'F21','F220','F228','F229','F230','F231','F232','F233','F238','F239', 'F24', 'F250','F251','F252','F258','F259','F28','F29','F300','F301','F302','F308','F309','F310','F311','F312','F313','F314','F315','F316','F317','F318','F319' | N = 13 |  |
| Treatment medication code.  Full cohort | Antipsychotics | Presence of antipsychotics in treatment medication code (f.20003). | N = 2 956 | Antipsychotics in Supplementary Table 5 |
| **Combine Above** | **Lifetime diagnosis of psychotic disorders** | | **6 727** |  |

**Supplementary Table 5.** List of antidepressants and antipsychotics used for defining symptoms of depression and probable diagnosis of psychotic disorders.

| **Antidepressants** | | **Antipsychotics** | |
| --- | --- | --- | --- |
| **UKB drug code** | **Drug name** | **UKB drug code** | **Drug name** |
| 1140879616 | amitriptyline | 1140868170 | prochlorperazine |
| 1140921600 | citalopram | 1140928916 | olanzapine |
| 1140879540 | fluoxetine | 1141152848 | quetiapine |
| 1140867878 | sertraline | 1140867444 | risperidone |
| 1140916282 | venlafaxine | 1140879658 | chlorpromazine |
| 1140909806 | dosulepin | 1140868120 | trifluoperazine |
| 1140867888 | paroxetine | 1141153490 | amisulpride |
| 1141152732 | mirtazapine | 1140867304 | sulpiride |
| 1141180212 | escitalopram | 1141152860 | seroquel |
| 1140879634 | trazodone | 1140867168 | haloperidol |
| 1140867876 | prozac | 1141195974 | aripiprazole |
| 1140882236 | seroxat | 1140867244 | stelazine |
| 1141190158 | cipralex | 1140867152 | depixol |
| 1141200564 | duloxetine | 1140909800 | flupentixol |
| 1140867726 | lofepramine | 1140867420 | clozapine |
| 1140879620 | clomipramine | 1140879746 | promazine |
| 1140867818 | nortriptyline | 1141177762 | risperdal |
| 1140879630 | imipramine | 1140867456 | modecate |
| 1140879628 | dothiepin | 1140867952 | fluanxol |
| 1141151946 | cipramil | 1140867150 | flupenthixol |
| 1140867948 | amitriptyline | 1141167976 | zyprexa |
| 1140867624 | prothiaden | 1140882100 | zuclopenthixol |
| 1140867756 | trimipramine | 1140867342 | clopixol |
| 1140867884 | lustral | 1140863416 | largactil |
| 1141151978 | reboxetine | 1141202024 | abilify |
| 1141152736 | zispin | 1140882098 | fluphenazine |
| 1141201834 | cymbalta | 1140867184 | haldol |
| 1140867690 | anafranil | 1140867092 | serenace |
| 1140867640 | doxepin | 1140882320 | clozaril |
| 1140867920 | moclobemide | 1140910358 | cpz |
| 1140867850 | phenelzine | 1140867208 | perphenazine |
| 1140879544 | fluvoxamine | 1140909802 | levomepromazine |
| 1141200570 | yentreve | 1140867134 | pericyazine |
| 1140867934 | triptafen | 1140867306 | dolmatil |
| 1140867758 | surmontil | 1140867210 | fentazin |
| 1140867914 | tranylcypromine | 1140867398 | fluphenazine |
| 1140867820 | allegron | 1140867078 | benperidol |
| 1141151982 | edronax | 1140867218 | pimozide |
| 1140882244 | molipaxin | 1141201792 | zaponex |
| 1140879556 | mianserin | 1141200458 | denzapine |
| 1140867852 | nardil | 1140867136 | neulactil |
| 1140867860 | faverin | 1140879750 | thioridazine |
| 1140917460 | nefazodone | 1140867180 | dozic |
| 1140867938 | amitriptyline+chlordiazepoxide | 1140867546 | fluspirilene |
| 1140867856 | isocarboxazid | 1140928260 | panadeine |
| 1140867922 | manerix | 1140927956 | sertindole |
| 1140910820 | maoi |  |  |
| 1140882312 | sinequan |  |  |
| 1140867944 | tranylcypromine+trifluoperazine |  |  |
| 1140867784 | ludiomil |  |  |
| 1140867812 | norval |  |  |
| 1140867668 | tryptizol |  |  |
| 1140867940 | fluphenazine |  |  |

**Supplementary Table 6.** Number of individuals excluded as part of the quality control of genetic data.

| **Phenotype** | **N with phenotype available** | **Non-European ancestry / Genetic data not available / QC exclusion** | **Related individuals excluded** | **Sample size after quality control** |
| --- | --- | --- | --- | --- |
| **Maternal smoking around birth** | 432 897 | 37 159 | 26 458 | No = 255 386  Yes = 113 894  **Total = 369 280** |
| **Smoking status** | 499 590 | 40 842 | 33 342 | Never = 229 221  Former = 151 605  Current = 44 580  **Total = 425 406** |
| **Pack smoked/year** | 150 950 | 8 575 | 4 620 | Mean = 23.5  Median = 19.2  **Total = 137 755** |
| **Any psychotic experience** | 157 357 | 8 010 | 4 233 | *Controls* = 137 695  *Cases* = 7 419  **Total = 145 114** |
| **Hallucinations** | 157 025 | 7 978 | 4 229 | *Controls* = 138 654  *Cases* = 6 164  **Total = 144 818** |
| **Delusions** | 157 249 | 7 992 | 4 233 | *Controls* = 143 145  *Cases* = 1 879  **Total = 145 024** |

**Supplementary Table 7.** Co-occurrence of smoking behaviours and psychotic experiences after quality control.

|  | | **Participants with genomic data after QC** | | | | | |
| --- | --- | --- | --- | --- | --- | --- | --- |
|  |  | **N** | **Maternal Smoking =Yes (%)** | **Current Smokers (%)** | **Former Smokers (%)** | **Never smoked (%)** | **Mean packs per year (SE)** |
| **Any psychotic experience** | *Cases* | 7 419 | 2 126  (28.7%) | 775  (10.4%) | 2 732  (36.8%) | 3 616  (48.7%) | 22.0  (0.36) |
|  | *Controls* | 137 695 | 33 769  (24.5%) | 9 139  (6.6%) | 46 692  (33.9%) | 75 547  (54.9%) | 20.0  (0.09) |
| **Delusions** | *Cases* | 1 879 | 547  (29.1%) | 237  (12.6%) | 693  (36.9%) | 872  (46.4%) | 23.0  (0.67) |
|  | *Controls* | 143 145 | 35 330  (24.7%) | 9 667  (6.8%) | 48 695  (34.0%) | 78 250  (54.7%) | 20.1  (0.08) |
| **Hallucinations** | *Cases* | 6 164 | 1 778  (28.8%) | 647  (10.5%) | 2 254  (36.6%) | 3 016  (48.9%) | 21.7  (0.38) |
|  | *Controls* | 138 654 | 34 048  (24.6%) | 9 241  (6.7%) | 47 060  (33.9%) | 76 002  (54.8%) | 20.0  (0.09) |

**Supplementary Table 8**. Interaction effects between PRSs and smoking status (never vs former vs current) on psychotic experiences (delusions, hallucinations or any of these two). Interaction effects are on the multiplicative (OR) and additive scale (β). Results are reported at the p-value threshold with the highest R^2^ in main PRS analyses (*p*= 0.2).

| **Predictor** | ***Multiplicative*** *(logistic regression)* | | | ***Additive*** *(linear regression)* | | |
| --- | --- | --- | --- | --- | --- | --- |
|  | **OR** | **95%CI** | ***p*** | **β** | **Std. Error** | ***p*** |
| ***Any psychotic experience*** | | | | | | |
| PRS_SCZ_ at P-value threshold = 0.2 | 1.29 | 1.07-1.56 | 0.008174 | 1.182 x 10^-2^ | 4.970 x 10^-3^ | 0.017401 |
| Former vs never smokers | 0.91 | 0.71-1.17 | 0.478631 | -5.035 x 10^-3^ | 6.408 x 10^-3^ | 0.432053 |
| Current vs never smokers | 1.43 | 0.98-2.07 | 0.059050 | 2.444 x 10^-2^ | 1.111 x 10^-2^ | 0.027825 |
| PRS_SCZ_ x former smokers | 1.04 | 0.95-1.14 | 0.363730 | 3.917 x 10^-3^ | 2.266 x 10^-3^ | 0.083958 |
| PRS_SCZ_ x current smokers | 0.98 | 0.85-1.13 | 0.779777 | 3.967 x 10^-3^ | 4.244 x 10^-3^ | 0.349915 |
| PRS_DEP_ at P-value threshold = 0.2 | 1.33 | 1.17-1.50 | 8.09 x 10^-6^ | 1.452 x 10^-2^ | 3.272 x 10^-3^ | 9.16 x 10^-6^ |
| Former vs never smokers | 1.01 | 0.96-1.08 | 0.450368 | -5.670 x 10^-3^ | 6.409 x 10^-3^ | 0.376358 |
| Current vs never smokers | 0.99 | 0.91-1.09 | 0.061811 | 2.399 x 10^-2^ | 1.111 x 10^-2^ | 0.030779 |
| PRS_DEP_ x former smokers | 1.02 | 0.96-1.08 | 0.572737 | 1.905 x 10^-3^ | 1.404 x 10^-3^ | 0.174883 |
| PRS_DEP_ x current smokers | 0.99 | 0.91-1.09 | 0.888421 | 3.036 x 10^-3^ | 2.635 x 10^-3^ | 0.249182 |
| PRS_ADHD_ at P-value threshold = 0.2 | 1.12 | 0.99-1.27 | 0.06544 | 5.499 x 10^-3^ | 3.255 x 10^-3^ | 0.09116 |
| Former vs never smokers | 0.91 | 0.71-1.17 | 0.46853 | -5.351 x 10^-3^ | 6.418 x 10^-3^ | 0.40441 |
| Current vs never smokers | 1.41 | 0.96-2.03 | 0.07427 | 2.388 x 10^-2^ | 1.113 x 10^-2^ | 0.03189 |
| PRS_ADHD_ x former smokers | 1.00 | 0.95-1.05 | 0.96207 | 3.795 x 10^-4^ | 1.356 x 10^-3^ | 0.77960 |
| PRS_ADHD_ x current smokers | 1.04 | 0.96-1.14 | 0.29890 | 5.010 x 10^-3^ | 2.512 x 10^-3^ | 0.04613 |
| PRS_BP_ at P-value threshold = 0.2 | 1.14 | 0.99-1.31 | 0.07571 | 5.925 x 10^-3^ | 3.690 x 10^-3^ | 0.10829 |
| Former vs never smokers | 0.93 | 0.72-1.19 | 0.54050 | -4.735 x 10^-3^ | 6.408 x 10^-3^ | 0.46000 |
| Current vs never smokers | 1.44 | 0.99-2.08 | 0.05486 | 2.493 x 10^-2^ | 1.111 x 10^-2^ | 0.02478 |
| PRS_BP_ x former smokers | 0.99 | 0.93-1.05 | 0.67545 | 2.533 x 10^-4^ | 1.571 x 10^-3^ | 0.87192 |
| PRS_BP_ x current smokers | 0.99 | 0.90-1.10 | 0.93611 | 2.887 x 10^-3^ | 2.894 x 10^-3^ | 0.31850 |
| ***Hallucinations*** | | | | | | |
| PRS_SCZ_ at P-value threshold = 0.2 | 1.24 | 1.00-1.53 | 0.046345 | 8.176 x 10^-3^ | 4.559 x 10^-3^ | 0.072928 |
| Former vs never smokers | 0.97 | 0.74-1.28 | 0.846827 | -1.533 x 10^-3^ | 5.881 x 10^-3^ | 0.794277 |
| Current vs never smokers | 1.27 | 0.83-1.92 | 0.261589 | 1.296 x 10^-2^ | 1.019 x 10^-2^ | 0.203709 |
| PRS_SCZ_ x former smokers | 1.04 | 0.94-1.15 | 0.435497 | 2.899 x 10^-3^ | 2.080 x 10^-3^ | 0.163324 |
| PRS_SCZ_ x current smokers | 0.95 | 0.81-1.11 | 0.496077 | 4.676 x 10^-4^ | 3.894 x 10^-3^ | 0.904404 |
| PRS_DEP_ at P-value threshold = 0.2 | 1.32 | 1.15-1.52 | 6.62 x 10^-5^ | 1.159 x 10^-2^ | 3.003 x 10^-3^ | 0.000113 |
| Former vs never smokers | 0.97 | 0.74-1.27 | 0.829991 | -2.036 x 10^-3^ | 5.882 x 10^-3^ | 0.729192 |
| Current vs never smokers | 1.28 | 0.84-1.94 | 0.240235 | 1.259 x 10^-2^ | 1.019 x 10^-2^ | 0.216444 |
| PRS_DEP_ x former smokers | 1.00 | 0.94-1.06 | 0.972918 | 8.890 x 10^-4^ | 1.289 x 10^-3^ | 0.490336 |
| PRS_DEP_ x current smokers | 0.92 | 0.83-1.02 | 0.099946 | -1.769 x 10^-3^ | 2.419 x 10^-3^ | 0.464589 |
| PRS_ADHD_ at P-value threshold = 0.2 | 1.13 | 0.98-1.29 | 0.093425 | 4.513 x 10^-3^ | 2.986 x 10^-3^ | 0.13077 |
| Former vs never smokers | 0.97 | 0.74-1.28 | 0.853878 | -1.712 x 10^-3^ | 5.890 x 10^-3^ | 0.77134 |
| Current vs never smokers | 1.26 | 0.82-1.90 | 0.276445 | 1.254 x 10^-2^ | 1.021 x 10^-2^ | 0.21924 |
| PRS_ADHD_ x former smokers | 0.97 | 0.91-1.03 | 0.274782 | -9.471 x 10^-4^ | 1.244 x 10^-3^ | 0.44655 |
| PRS_ADHD_ x current smokers | 0.99 | 0.89-1.08 | 0.755347 | 1.041 x 10^-3^ | 2.306 x 10^-3^ | 0.65176 |
| PRS_BP_ at P-value threshold = 0.2 | 1.04 | 0.89-1.22 | 0.59818 | 1.198 x 10^-3^ | 3.385 x 10^-3^ | 0.723415 |
| Former vs never smokers | 0.98 | 0.75-1.29 | 0.90387 | -1.211 x 10^-3^ | 5.881 x 10^-3^ | 0.836856 |
| Current vs never smokers | 1.28 | 0.83-1.93 | 0.24741 | 1.336 x 10^-2^ | 1.019 x 10^-2^ | 0.189822 |
| PRS_BP_ x former smokers | 0.99 | 0.92-1.06 | 0.80120 | 1.743 x 10^-4^ | 1.442 x 10^-3^ | 0.903783 |
| PRS_BP_ x current smokers | 0.99 | 0.89-1.10 | 0.81744 | 1.224 x 10^-3^ | 2.657 x 10^-3^ | 0.645022 |
| ***Delusions*** | | | | | | |
| PRS_SCZ_ at P-value threshold = 0.2 | 1.37 | 0.94-1.98 | 0.0955 | 3.655 x 10^-3^ | 2.552 x 10^-3^ | 0.152147 |
| Former vs never smokers | 0.72 | 0.44-1.18 | 0.1970 | -4.435 x 10^-3^ | 3.291 x 10^-3^ | 0.177755 |
| Current vs never smokers | 2.08 | 1.09-3.76 | 0.0195 | 1.936 x 10^-2^ | 5.704 x 10^-3^ | 0.000687 |
| PRS_SCZ_ x former smokers | 1.02 | 0.85-1.22 | 0.8253 | 1.534 x 10^-3^ | 1.164 x 10^-3^ | 0.187645 |
| PRS_SCZ_ x current smokers | 0.98 | 0.75-1.28 | 0.8951 | 4.709 x 10^-3^ | 2.180 x 10^-3^ | 0.030753 |
| PRS_DEP_ at P-value threshold = 0.2 | 1.25 | 0.99-1.58 | 0.05919 | 3.862 x 10^-3^ | 1.681 x 10^-3^ | 0.021577 |
| Former vs never smokers | 0.72 | 0.44-1.16 | 0.18142 | -4.667 x 10^-3^ | 3.292 x 10^-3^ | 0.156301 |
| Current vs never smokers | 2.02 | 1.06-3.68 | 0.02576 | 1.917 x 10^-2^ | 5.703 x 10^-3^ | 0.000774 |
| PRS_DEP_ x former smokers | 0.72 | 0.44-1.16 | 0.12162 | 1.526 x 10^-3^ | 7.214 x 10^-4^ | 0.034431 |
| **PRS_DEP_ x current smokers** | 2.02 | 1.06-3.68 | 0.06714 | **5.176 x 10^-3^** | **1.354 x 10^-3^** | **0.000132** |
| PRS_ADHD_ at P-value threshold = 0.2 | 1.05 | 0.84-1.32 | 0.656602 | 6.638 x 10^-4^ | 1.672 x 10^-3^ | 0.691301 |
| Former vs never smokers | 0.73 | 0.45-1.18 | 0.201909 | -4.565 x 10^-3^ | 3.296 x 10^-3^ | 0.166047 |
| Current vs never smokers | 2.01 | 1.06-3.66 | 0.026040 | 1.926 x 10^-2^ | 5.714 x 10^-3^ | 0.000752 |
| PRS_ADHD_ x former smokers | 1.05 | 0.95-1.17 | 0.343882 | 7.083 x 10^-4^ | 6.964 x 10^-4^ | 0.309129 |
| **PRS_ADHD_ x current smokers** | 1.23 | 1.05-1.44 | 0.008462 | **5.004 x 10^-3^** | **1.290 x 10^-3^** | **0.000106** |
| PRS_BP_ at P-value threshold = 0.2 | 1.41 | 1.08-1.83 | 0.01095 | 4.388 x 10^-3^ | 1.895 x 10^-3^ | 0.020569 |
| Former vs never smokers | 0.75 | 0.46-1.21 | 0.24029 | -4.393 x 10^-3^ | 3.291 x 10^-3^ | 0.181861 |
| Current vs never smokers | 2.14 | 1.13-3.85 | 0.01458 | 1.955 x 10^-2^ | 5.703 x 10^-3^ | 0.000608 |
| PRS_BP_ x former smokers | 0.99 | 0.87-1.12 | 0.81740 | 5.200 x 10^-4^ | 8.069 x 10^-4^ | 0.519311 |
| PRS_BP_ x current smokers | 0.93 | 0.78-1.12 | 0.43936 | 1.492 x 10^-3^ | 1.487 x 10^-3^ | 0.315851 |

Note. All analyses included the first ten principal components, genotyping batch and the interaction between those and smoking status and PRS as covariates ^3^. Results from each of these covariates were not of interest to the study and are therefore not displayed. Also, due to the inclusion of interaction terms in this model, main effects should be interpreted as conditional on the interacting variables. The main effects of PRSs and smoking status on psychotic experiences are reported in Figure 2 and Table 2, respectively.

**Supplementary Table 9.** Number of individuals with psychotic disorders that were excluded.

| **Phenotype** | **Sample size after genetic QC** | **Individuals with psychotic disorders excluded** | **Final sample size** |
| --- | --- | --- | --- |
| **Lifetime delusions** | *Controls* = 143 145  *Cases* = 1 879  **Total = 145 024** | 1 388  *(458 cases + 1 547 controls)* | *Controls* = 141 598  *Cases* = 1 421  **Total = 143 019** |

**Supplementary Table 10**. Interaction effects between PRSs and smoking status (never vs former vs current) on delusions after excluding individuals with psychotic disorders. Interaction effects are on the additive scale (β). Results are reported at the p-value threshold with the highest R^2^ in main PRS analyses (*p* = 0.2).

| **Predictor** | ***Additive*** *(linear regression)* | | |
| --- | --- | --- | --- |
|  | **β** | **Std. Error** | ***p*** |
| PRS_DEP_ at P-value threshold = 0.2 | 2.437 x 10^-3^ | 1.490 x 10^-3^ | 0.10184 |
| Former vs never smokers | -4.555 x 10^-3^ | 2.905 x 10^-3^ | 0.11685 |
| Current vs never smokers | 8.096 x 10^-3^ | 5.053 x 10^-3^ | 0.10909 |
| PRS_DEP_ x former smokers | 1.408 x 10^-3^ | 6.357 x 10^-4^ | 0.02675 |
| **PRS_DEP_ x current smokers** | **3.740 x 10^-3^** | **1.200 x 10^-3^** | **0.00182** |
| PRS_ADHD_ at P-value threshold = 0.2 | 4.874 x 10^-4^ | 1.480 x 10^-3^ | 0.741882 |
| Former vs never smokers | -4.594 x 10^-3^ | 2.908 x 10^-3^ | 0.114222 |
| Current vs never smokers | 7.866 x 10^-3^ | 5.063 x 10^-3^ | 0.120272 |
| PRS_ADHD_ x former smokers | 8.980 x 10^-4^ | 6.135 x 10^-4^ | 0.143263 |
| **PRS_ADHD_ x current smokers** | **4.349 x 10^-3^** | **1.144 x 10^-3^** | **0.000143** |

Note. All analyses included the first ten principal components, genotyping batch and the interaction between those and smoking status and PRS as covariates ^3^. Results from each of these covariates were not of interest to the study and are therefore not displayed. Also, due to the inclusion of interaction terms in this model, main effects should be interpreted as conditional on the interacting variables. The main effects of PRSs and smoking status on psychotic experiences are reported in Figure 2 and Table 2, respectively.

**Supplementary Table 11**. Interaction effects between PRSs and maternal smoking on psychotic experiences (delusions, hallucinations or any of these two). Interaction effects are on the multiplicative (OR) and additive scale (β). Results are reported at the p-value threshold with the highest R^2^ in in main PRS analyses (*p* = 0.2).

| **Predictor** | ***Multiplicative*** *(logistic regression)* | | | ***Additive*** *(linear regression)* | | |
| --- | --- | --- | --- | --- | --- | --- |
|  | **OR** | **95%CI** | ***p*** | **β** | **Std. Error** | ***p*** |
| ***Any psychotic experience*** | | | | | | |
| PRS_SCZ_ at P-value threshold = 0.2 | 1.31 | 1.06-1.60 | 0.0100 | 1.264 x 10^-2^ | 5.264 x 10^-3^ | 0.0163 |
| Maternal smoking = YES | 1.40 | 1.08-1.81 | 0.0105 | 1.887 x 10^-2^ | 6.958 x 10^-3^ | 0.0067 |
| PRS_SCZ_ x Maternal smoking = YES | 0.95 | 0.86-1.04 | 0.2764 | -5.800 x 10^-4^ | 2.490 x 10^-3^ | 0.8158 |
| PRS_DEP_ at P-value threshold = 0.2 | 1.34 | 1.17-1.53 | 1.42 x 10^-5^ | 1.513 x 10^-2^ | 3.453 x 10^-3^ | 1.17 x 10^-5^ |
| Maternal smoking = YES | 1.36 | 1.05-1.76 | 0.0191 | 1.793 x 10^-2^ | 6.955 x 10^-3^ | 0.0099 |
| PRS_DEP_ x Maternal smoking = YES | 1.01 | 0.95-1.07 | 0.8002 | 1.906 x 10^-3^ | 1.547 x 10^-3^ | 0.2181 |
| PRS_ADHD_ at P-value threshold = 0.2 | 1.13 | 0.99-1.30 | 0.0665 | 5.993 x 10^-3^ | 3.455 x 10^-3^ | 0.0829 |
| Maternal smoking = YES | 1.39 | 1.07-1.79 | 0.0133 | 1.892 x 10^-2^ | 6.954 x 10^-3^ | 0.0065 |
| PRS_ADHD_ x Maternal smoking = YES | 0.97 | 0.92-1.03 | 0.3185 | -6.253 x 10^-4^ | 1.486 x 10^-3^ | 0.6740 |
| PRS_BP_ at P-value threshold = 0.2 | 1.20 | 1.03-1.39 | 0.01780 | 9.000 x 10^-3^ | 3.889 x 10^-3^ | 0.020665 |
| Maternal smoking = YES | 1.40 | 1.08-1.81 | 0.01031 | 1.927 x 10^-2^ | 6.954 x 10^-3^ | 0.005586 |
| PRS_BP_ x Maternal smoking = YES | 0.97 | 0.90-1.03 | 0.31508 | -7.971 x 10^-4^ | 1.723 x 10^-3^ | 0.643634 |
| ***Hallucinations*** | | | | | | |
| PRS_SCZ_ at P-value threshold = 0.2 | 1.23 | 0.98-1.55 | 0.0718 | 7.986 x 10^-3^ | 4.828 x 10^-3^ | 0.0981 |
| Maternal smoking = YES | 1.44 | 1.08-1.91 | 0.0131 | 1.651 x 10^-2^ | 6.381 x 10^-3^ | 0.0097 |
| PRS_SCZ_ x Maternal smoking = YES | 0.97 | 0.88-1.08 | 0.6274 | 4.919 x 10^-4^ | 2.284 x 10^-3^ | 0.8295 |
| PRS_DEP_ at P-value threshold = 0.2 | 1.34 | 1.15-1.55 | 0.0001 | 1.187 x 10^-2^ | 3.167 x 10^-3^ | 0.0002 |
| Maternal smoking = YES | 1.41 | 1.06-1.86 | 0.0190 | 1.590 x 10^-2^ | 6.378 x 10^-3^ | 0.0127 |
| PRS_DEP_ x Maternal smoking = YES | 1.00 | 0.93-1.07 | 0.9412 | 1.116 x 10^-3^ | 1.420 x 10^-3^ | 0.4319 |
| PRS_ADHD_ at P-value threshold = 0.2 | 1.11 | 0.96-1.29 | 0.1683 | 3.863 x 10^-3^ | 3.169 x 10^-3^ | 0.2228 |
| Maternal smoking = YES | 1.43 | 1.07-1.89 | 0.0150 | 1.672 x 10^-2^ | 6.376 x 10^-3^ | 0.0087 |
| PRS_ADHD_ x Maternal smoking = YES | 0.99 | 0.93-1.06 | 0.7802 | 4.108 x 10^-4^ | 1.364 x 10^-3^ | 0.7632 |
| PRS_BP_ at P-value threshold = 0.2 | 1.11 | 0.94-1.31 | 0.208525 | 4.243 x 10^-3^ | 3.567 x 10^-3^ | 0.234232 |
| Maternal smoking = YES | 1.44 | 1.08-1.91 | 0.012634 | 1.691 x 10^-2^ | 6.377 x 10^-3^ | 0.008026 |
| PRS_BP_ x Maternal smoking = YES | 0.96 | 0.89-1.04 | 0.311359 | -1.053 x 10^-3^ | 1.580 x 10^-3^ | 0.505132 |
| ***Delusions*** | | | | | | |
| PRS_SCZ_ at P-value threshold = 0.2 | 1.39 | 0.93-2.03 | 0.1031 | 4.255 x 10^-3^ | 2.695 x 10^-3^ | 0.1144 |
| Maternal smoking = YES | 1.19 | 0.72-1.92 | 0.4920 | 4.591 x 10^-3^ | 3.562 x 10^-3^ | 0.1975 |
| PRS_SCZ_ x Maternal smoking = YES | 0.86 | 0.71-1.05 | 0.1319 | -7.001 x 10^-4^ | 1.275 x 10^-3^ | 0.5828 |
| PRS_DEP_ at P-value threshold = 0.2 | 1.33 | 1.04-1.69 | 0.0243 | 4.733 x 10^-3^ | 1.768 x 10^-3^ | 0.0074 |
| Maternal smoking = YES | 1.13 | 0.69-1.83 | 0.6161 | 4.406 x 10^-3^ | 3.561 x 10^-3^ | 0.2160 |
| PRS_DEP_ x Maternal smoking = YES | 1.03 | 0.92-1.16 | 0.5867 | 1.124 x 10^-3^ | 7.925 x 10^-4^ | 0.1563 |
| PRS_ADHD_ at P-value threshold = 0.2 | 1.10 | 0.85-1.40 | 0.4758 | 1.199 x 10^-3^ | 1.769 x 10^-3^ | 0.4978 |
| Maternal smoking = YES | 1.16 | 0.71-1.88 | 0.5400 | 4.647 x 10^-3^ | 3.560 x 10^-3^ | 0.1918 |
| PRS_ADHD_ x Maternal smoking = YES | 0.94 | 0.85-1.06 | 0.3620 | -4.894 x 10^-4^ | 7.611 x 10^-4^ | 0.5202 |
| PRS_BP_ at P-value threshold = 0.2 | 1.45 | 1.09-1.90 | 0.00895 | 4.914 x 10^-3^ | 1.991 x 10^-3^ | 0.01359 |
| Maternal smoking = YES | 1.20 | 0.74-1.93 | 0.44791 | 4.764 x 10^-3^ | 3.560 x 10^-3^ | 0.18083 |
| PRS_BP_ x Maternal smoking = YES | 0.90 | 0.79-1.03 | 0.12359 | -8.891 x 10^-4^ | 8.822 x 10^-4^ | 0.31355 |

Note. All analyses included the first ten principal components, genotyping batch and the interaction between those and maternal smoking and PRS as covariates ^3^. Results from each of these covariates were not of interest to the study and are therefore not displayed. Also, due to the inclusion of interaction terms in this model, main effects should be interpreted as conditional on the interacting variables. The main effects of PRSs and maternal smoking are reported in Figure 2 and Table 2, respectively.

**Supplementary Table 12**. Interaction effects between PRSs and the number of packs smoked per year on psychotic experiences (delusions, hallucinations or any of these two). Interaction effects are on the multiplicative (OR) and additive scale (β). Results are reported at the p-value threshold with the highest R^2^ in in main PRS analyses (p-value threshold = 0.2).

| **Predictor** | ***Multiplicative*** *(logistic regression)* | | | ***Additive*** *(linear regression)* | | |
| --- | --- | --- | --- | --- | --- | --- |
|  | **OR** | **95%CI** | ***p*** | **β** | **Std. Error** | ***p*** |
| ***Any psychotic experience*** | | | | | | |
| PRS_SCZ_ at P-value threshold = 0.2 | 1.50 | 1.07-2.08 | 0.0165 | 2.161 x 10^-2^ | 1.013 x 10^-2^ | 0.0329 |
| Number of packs per year | 1.02 | 1.01-1.03 | 0.0004 | 1.391 x 10^-3^ | 3.754 x 10^-4^ | 0.0002 |
| PRS_SCZ_ x Number of packs per year | 1.00 | 1.00-1.01 | 0.6017 | 1.651 x 10^-4^ | 1.344 x 10^-4^ | 0.2195 |
| PRS_DEP_ at P-value threshold = 0.2 | 1.32 | 1.06-1.65 | 0.0141 | 1.545 x 10^-2^ | 6.827 x 10^-3^ | 0.0237 |
| Number of packs per year | 1.02 | 1.01-1.03 | 0.0012 | 1.322 x 10^-3^ | 3.759 x 10^-4^ | 0.0004 |
| PRS_DEP_ x Number of packs per year | 1.00 | 0.99-1.00 | 0.1972 | 1.809 x 10^-4^ | 8.292 x 10^-5^ | 0.0291 |
| PRS_ADHD_ at P-value threshold = 0.2 | 1.28 | 1.04-1.57 | 0.0185 | 1.459 x 10^-2^ | 6.523 x 10^-3^ | 0.0253 |
| Number of packs per year | 1.02 | 1.01-1.03 | 0.0008 | 1.320 x 10^-3^ | 3.772 x 10^-4^ | 0.0005 |
| PRS_ADHD_ x Number of packs per year | 1.00 | 0.996-1.002 | 0.6109 | -1.326 x 10^-5^ | 8.202 x 10^-5^ | 0.8716 |
| PRS_BP_ at P-value threshold = 0.2 | 1.22 | 0.94-1.56 | 0.1268 | 1.048 x 10^-2^ | 7.628 x 10^-3^ | 0.1696 |
| Number of packs per year | 1.02 | 1.01-1.03 | 0.0008 | 1.372 x 10^-3^ | 3.757 x 10^-4^ | 0.0002 |
| PRS_BP_ x Number of packs per year | 1.00 | 0.99-1.00 | 0.4343 | 1.269 x 10^-4^ | 9.129 x 10^-5^ | 0.1645 |
| ***Hallucinations*** | | | | | | |
| PRS_SCZ_ at P-value threshold = 0.2 | 1.54 | 1.07-2.19 | 0.0172 | 2.032 x 10^-2^ | 9.307 x 10^-3^ | 0.0290 |
| Number of packs per year | 1.01 | 1.00-1.03 | 0.0219 | 8.223 x 10^-4^ | 3.452 x 10^-4^ | 0.0172 |
| PRS_SCZ_ x Number of packs per year | 1.00 | 1.00-1.01 | 0.2756 | 2.006 x 10^-4^ | 1.235 x 10^-4^ | 0.1045 |
| PRS_DEP_ at P-value threshold = 0.2 | 1.22 | 0.95-1.55 | 0.1109 | 9.337 x 10^-3^ | 6.282 x 10^-3^ | 0.1372 |
| Number of packs per year | 1.01 | 1.00-1.02 | 0.0361 | 7.685 x 10^-4^ | 3.457 x 10^-4^ | 0.0262 |
| PRS_DEP_ x Number of packs per year | 1.00 | 0.99-1.00 | 0.1759 | 1.443 x 10^-4^ | 7.623 x 10^-5^ | 0.0584 |
| PRS_ADHD_ at P-value threshold = 0.2 | 1.24 | 0.98-1.55 | 0.0576 | 1.078 x 10^-2^ | 5.996 x 10^-3^ | 0.0723 |
| Number of packs per year | 1.01 | 1.00-1.03 | 0.0265 | 7.746 x 10^-4^ | 3.468 x 10^-4^ | 0.0255 |
| PRS_ADHD_ x Number of packs per year | 1.00 | 0.996-1.001 | 0.4402 | -5.297 x 10^-5^ | 7.537 x 10^-5^ | 0.4822 |
| PRS_BP_ at P-value threshold = 0.2 | 1.18 | 0.89-1.55 | 0.2444 | 6.852 x 10^-3^ | 7.012 x 10^-3^ | 0.3285 |
| Number of packs per year | 1.01 | 1.00-1.02 | 0.0268 | 8.033 x 10^-4^ | 3.454 x 10^-4^ | 0.0201 |
| PRS_BP_ x Number of packs per year | 1.00 | 0.99-1.00 | 0.5781 | 7.316 x 10^-5^ | 8.388 x 10^-5^ | 0.3831 |
| ***Delusions*** | | | | | | |
| PRS_SCZ_ at P-value threshold = 0.2 | 1.77 | 0.94-3.23 | 0.0704 | 8.805 x 10^-3^ | 5.386 x 10^-3^ | 0.1021 |
| Number of packs per year | 1.01 | 0.99-1.03 | 0.1415 | 3.336 x 10^-4^ | 1.997 x 10^-4^ | 0.0949 |
| PRS_SCZ_ x Number of packs per year | 0.99 | 0.99-1.00 | 0.2093 | -2.547 x 10^-5^ | 7.153 x 10^-5^ | 0.7218 |
| PRS_DEP_ at P-value threshold = 0.2 | 1.52 | 1.04-2.18 | 0.0271 | 7.913 x 10^-3^ | 3.631 x 10^-3^ | 0.0293 |
| Number of packs per year | 1.01 | 0.99-1.03 | 0.1615 | 3.100 x 10^-4^ | 2.000 x 10^-4^ | 0.1212 |
| PRS_DEP_ x Number of packs per year | 1.00 | 0.99-1.00 | 0.9568 | 4.754 x 10^-5^ | 4.413 x 10^-5^ | 0.2813 |
| PRS_ADHD_ at P-value threshold = 0.2 | 1.23 | 0.84-1.75 | 0.2817 | 4.032 x 10^-3^ | 3.468 x 10^-3^ | 0.2450 |
| Number of packs per year | 1.01 | 0.99-1.03 | 0.1672 | 3.069 x 10^-4^ | 2.006 x 10^-4^ | 0.1261 |
| PRS_ADHD_ x Number of packs per year | 1.00 | 1.00-1.01 | 0.8026 | 4.142 x 10^-5^ | 4.362 x 10^-5^ | 0.3424 |
| PRS_BP_ at P-value threshold = 0.2 | 1.51 | 0.96-2.37 | 0.0727 | 5.829 x 10^-3^ | 4.056 x 10^-3^ | 0.1507 |
| Number of packs per year | 1.01 | 0.99-1.03 | 0.1581 | 3.197 x 10^-4^ | 1.999 x 10^-4^ | 0.1096 |
| PRS_BP_ x Number of packs per year | 0.99 | 0.99-1.00 | 0.4601 | 5.345 x 10^-6^ | 4.856 x 10^-5^ | 0.9123 |

Note. All analyses included the first ten principal components, genotyping batch and the interaction between those and the number of packs smoked per year and PRS as covariates ^3^. Results from each of these covariates were not of interest to the study and are therefore not displayed. Also, due to the inclusion of interaction terms in this model, main effects should be interpreted as conditional on the interacting variables. The main effects of PRSs and the number of packs smoked per year are reported in Figure 2 and Table 2, respectively.


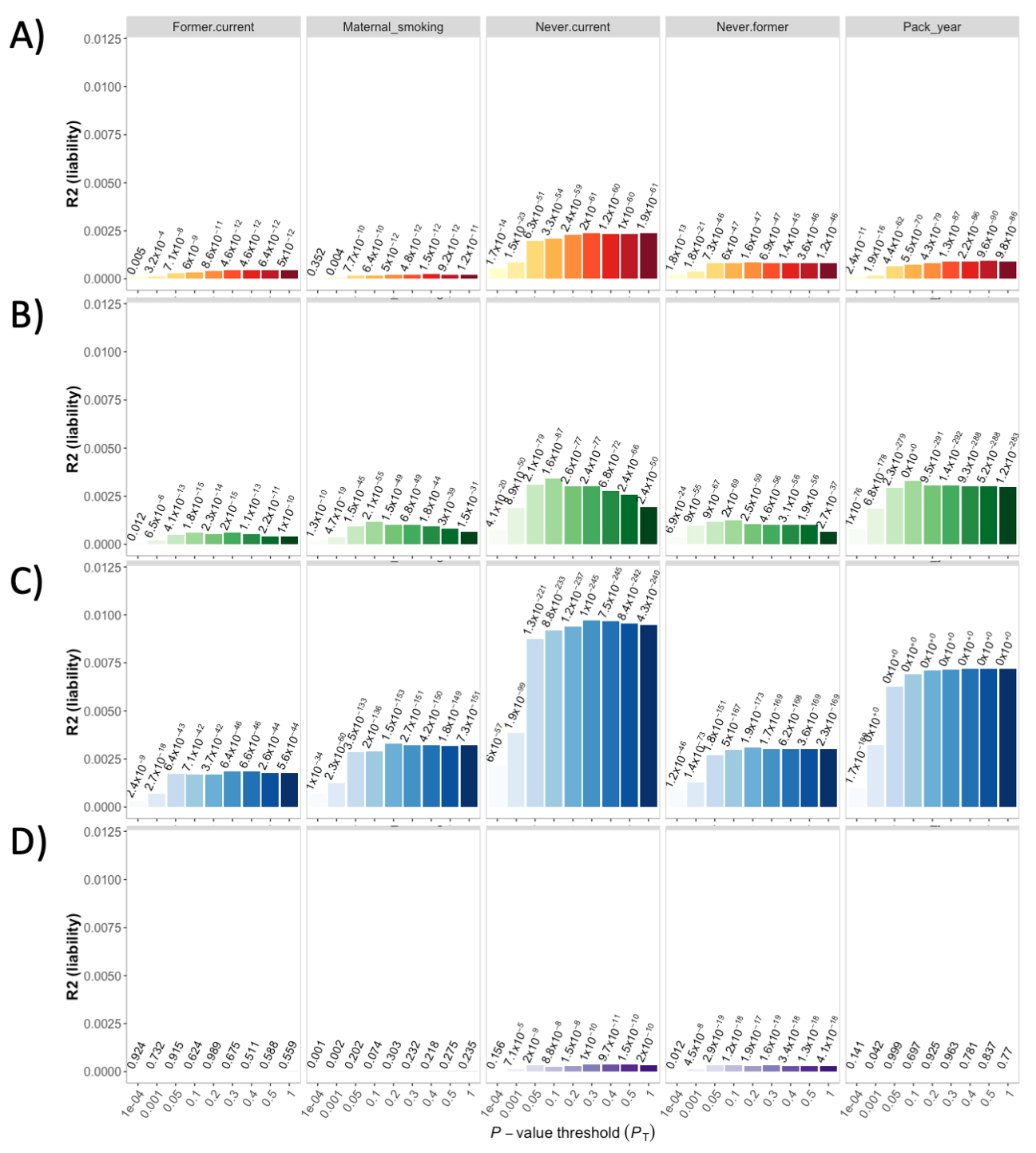


**Supplementary Figure 1:** Prediction of smoking behaviours (former vs current smoker, never vs current smoker, never vs former smoker, and maternal smoking around the participants’ birth) in UKB using PRSs for schizophrenia (Panel A), depression (Panel B), ADHD (Panel C) and bipolar disorder (Panel D). Each panel is divided into types of smoking behaviour. Logistic regression models were calculated using PRSice-2 to assess the association between the PRSs and each smoking behaviour. As the number of packs smoked per year represent count data, Poisson regression models were constructed in R to assess the association between the PRSs and number of packs smoked per year. The Y axis represents variance of psychotic experience explained by the PRS in the liability scale (Nagelkerke R^2^). The X axis represents different genetic association p-value thresholds used to build the PRS. The p-values of the association between the PRSs and psychotic experiences are listed above of the bars.

**
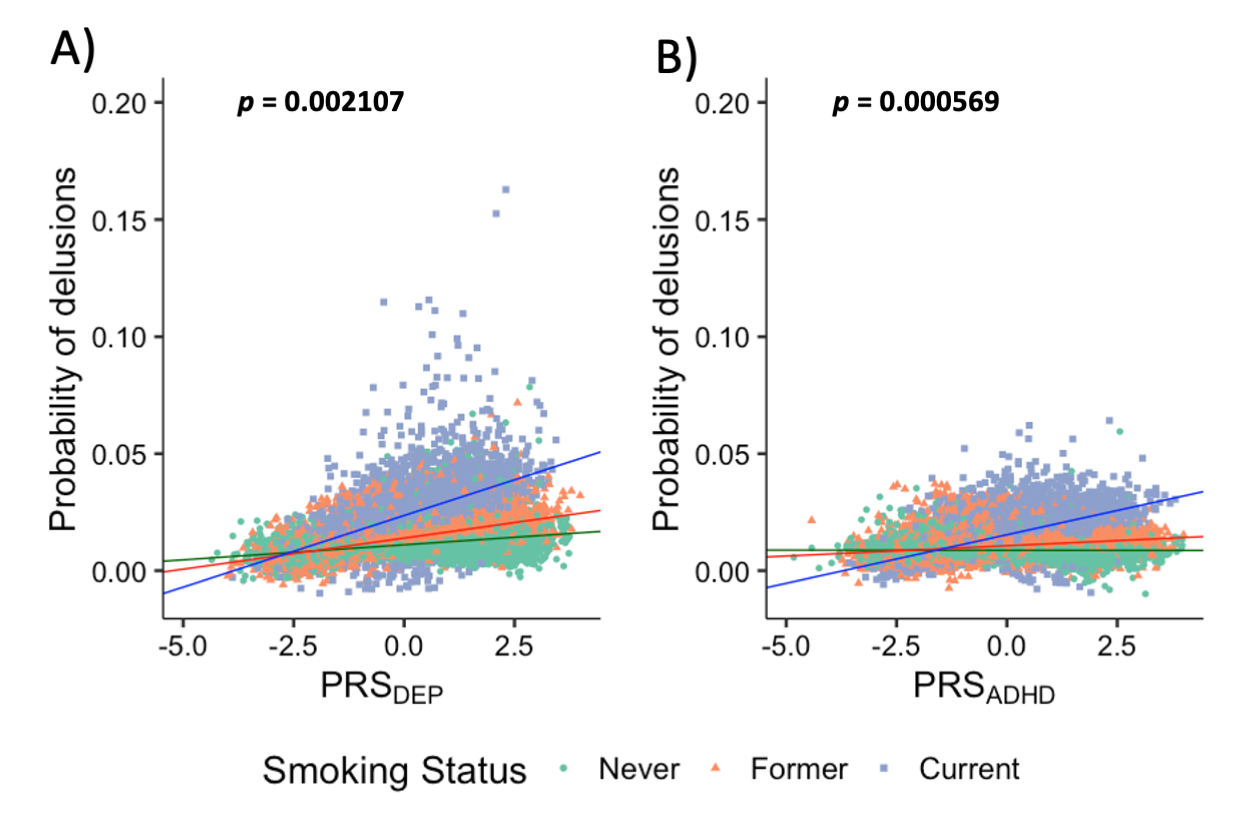
**

**Supplementary Figure 2**. Sensitivity analyses, excluding individuals with diagnosis of psychotic disorders. Figure shows associations between PRSs for A) depression and B) ADHD, and occurrence of delusions. Current smokers are shown as blue squares, former smokers as orange triangles, and never smokers as green circles. P-values for the PRS x smoking status interactions are shown on the upper part of each plot.

**Polygenic score approach**

We calculated polygenic risk scores (PRSs) using summary statistics from GWAS meta-analyses for depression ^4^, ADHD ^5^, schizophrenia ^6^ and bipolar disorder ^7^.

**a) Depression**: A recent GWAS meta-analysis including 246 363 cases and 561 190 controls identified 102 significant independent loci associated with depression ^4^. Because the original study included UKB data, we conducted a new meta-analysis excluding individuals from UKB to avoid biases due to sample overlap. The resulting meta-analysis contained 118 811 cases and 327 427 controls and identified 23 independent loci.

b) **ADHD**: GWAS meta-analysis of 20 183 ADHD cases and 35 191 controls identified 12 independent loci surpassing genome-wide significance ^5^. Only the European ancestry meta-analysis (19 099 cases and 34 194 controls) was used to calculate the genetic scores.

c) **Schizophrenia**: Results from the second Psychiatric Genomics Consortium (PGC) schizophrenia mega-analysis included up to 36 989 cases and 113 075 controls and identified 108 schizophrenia associated genetic loci ^6^.

d) **Bipolar disorder**: Results from the second GWAS of the PGC Bipolar Disorder Working Group included 20 352 cases and 31 358 controls of European descent, and identified 19 genome-wide significant loci ^7^.

The software PRSice2 (<https://choishingwan.github.io/PRSice/>) was used to calculate the PRSs ^8^. For computational reasons, directly genotyped variants were used in the UKB dataset. Preliminary analyses demonstrated that due to clumping of variants, results from genotyped vs imputed data are similar (not shown).

Ten p-value thresholds were used to compute the scores: 0.00000005; 0.000001; 0.0001; 0.001; 0.01; 0.05; 0.1; 0.2; 0.5 and 1. SNPs were selected if they had imputation INFO scores > 0,3 (for bipolar disorder), > 0.6 (for depression), and > 0.7 (for SCZ and ADHD), minor allele frequency > 0.01 (for ADHD, bipolar disorder and schizophrenia) or > 0.005 (for depression), and low linkage disequilibrium to each other (r^2^ < 0.1 within 250kb to both ends of the index SNP). For the dataset from UKB, we selected SNPs with a 90% genotyping rate and minor allele frequency >0.05. The PRSs were calculated under the additive model and standardised to a mean of zero and standard deviation of one to aid interpretation of results. Nagelkerke R^2^ was used to calculate the variance explained by the PRSs. However this metric suffers from bias when the proportion for cases controls is not reflective of the case population prevalence ^9^. Since there is evidence of a healthy volunteer selection bias in the UKB cohort ^10^, ascertainment biases of the R^2^ were adjusted in PRSice by providing population prevalence information for the binary phenotypes. Population prevalences were obtained from World Health Organisation World Mental Health Surveys ^11^: Lifetime prevalence of ever having a psychotic experience was set as 5.8%, hallucinations as 5.2% and delusional experiences as 1.3%. For maternal smoking, population prevalence was defined as [Ncases]/[Ntotal completing MHQ] and sample prevalence was defined as [Ncases/(Ncases + Ncontrols)]. This estimate was calculated as previously described ^12^ and might represent better the prevalence of smoking during pregnancy 50 years ago. Significance for the PRS association was declared at the p-value threshold of 0.005 (Bonferroni correction based on 9 independent tests, one for each p-value threshold analysed).

**PRS power calculation**

We performed power calculations for the polygenic analysis at each p-value threshold using the R package AVENGEME ^13^. Models assumed SNP heritability of 0.216 for ADHD ^5^, 0.33 for schizophrenia ^6^, 0.20 for bipolar disorder ^7^, and 0.085 for depression ^14^. The lifetimes prevalence used were 5% for ADHD ^15^, 0.87% for schizophrenia ^16^, 16.2% for depression ^17^, 1% for bipolar disorder ^18^, 5.8% for ever having a psychotic experience, 5.2% for hallucinations and 1.3% for delusions ^11^. The models used for power calculation assumed that the markers are independent.

Since all the PRS analyses were cross-trait, four hypothetical scenarios were tested, comparing change in statistical power when covariance between genetic effects in training and test samples were 25%, 50%, 75% and 100%.

All the PRS analyses performed under all the hypothetical scenarios gave > 95% power.

**SUPPLEMENTARY REFERENCES**

1. Davis, K. A. S. *et al.* Mental health in UK Biobank - development, implementation and results from an online questionnaire completed by 157 366 participants: a reanalysis. *BJPsych Open* **6**, e18 (2020).

2. Kessler, R. C., Andrews, G., Mroczek, D., Ustun, B. & Wittchen, H.-U. The World Health Organization Composite International Diagnostic Interview short-form (CIDI-SF). *International Journal of Methods in Psychiatric Research* **7**, 171–185 (1998).

3. Keller, M. C. Gene × Environment Interaction Studies Have Not Properly Controlled for Potential Confounders: The Problem and the (Simple) Solution. *Biological Psychiatry* **75**, 18–24 (2014).

4. Wray, N. R. *et al.* Genome-wide association analyses identify 44 risk variants and refine the genetic architecture of major depression. *Nat Genet* **50**, 668–681 (2018).

5. Demontis, D. *et al.* Discovery of the first genome-wide significant risk loci for attention deficit/hyperactivity disorder. *Nat Genet* **51**, 63–75 (2019).

6. Ripke, S. *et al.* Biological insights from 108 schizophrenia-associated genetic loci. *Nature* **511**, 421–427 (2014).

7. Stahl, E. A. *et al.* Genome-wide association study identifies 30 loci associated with bipolar disorder. *Nat. Genet.* **51**, 793–803 (2019).

8. Choi, S. W. & O’Reilly, P. F. PRSice-2: Polygenic Risk Score software for biobank-scale data. *Gigascience* **8**, (2019).

9. Lee, S. H., Wray, N. R., Goddard, M. E. & Visscher, P. M. Estimating missing heritability for disease from genome-wide association studies. *Am. J. Hum. Genet.* **88**, 294–305 (2011).

10. Fry, A. *et al.* Comparison of Sociodemographic and Health-Related Characteristics of UK Biobank Participants With Those of the General Population. *Am J Epidemiol* **186**, 1026–1034 (2017).

11. McGrath, J. J. *et al.* Psychotic Experiences in the General Population: A Cross-National Analysis Based on 31,261 Respondents From 18 Countries. *JAMA Psychiatry* **72**, 697–705 (2015).

12. Hodgson, K. *et al.* Cannabis use, depression and self-harm: phenotypic and genetic relationships. *bioRxiv* 549899 (2019) doi:10.1101/549899.

13. Palla, L. & Dudbridge, F. A Fast Method that Uses Polygenic Scores to Estimate the Variance Explained by Genome-wide Marker Panels and the Proportion of Variants Affecting a Trait. *The American Journal of Human Genetics* **97**, 250–259 (2015).

14. Howard, D. M. *et al.* Genome-wide association study of depression phenotypes in UK Biobank identifies variants in excitatory synaptic pathways. *Nat Commun* **9**, 1–10 (2018).

15. Polanczyk, G., de Lima, M. S., Horta, B. L., Biederman, J. & Rohde, L. A. The worldwide prevalence of ADHD: a systematic review and metaregression analysis. *Am J Psychiatry* **164**, 942–948 (2007).

16. Perälä, J. *et al.* Lifetime prevalence of psychotic and bipolar I disorders in a general population. *Arch. Gen. Psychiatry* **64**, 19–28 (2007).

17. Kessler, R. C. *et al.* The epidemiology of major depressive disorder: results from the National Comorbidity Survey Replication (NCS-R). *JAMA* **289**, 3095–3105 (2003).

18. Ferrari, A. J. *et al.* The prevalence and burden of bipolar disorder: findings from the Global Burden of Disease Study 2013. *Bipolar Disord* **18**, 440–450 (2016).
